# Distinct Responses of Leaf Traits to Environment and Phylogeny Between Herbaceous and Woody Angiosperm Species in China

**DOI:** 10.3389/fpls.2021.799401

**Published:** 2021-12-07

**Authors:** Nannan An, Nan Lu, Bojie Fu, Mengyu Wang, Nianpeng He

**Affiliations:** ^1^State Key Laboratory of Urban and Regional Ecology, Research Center for Eco-Environmental Sciences, Chinese Academy of Sciences, Beijing, China; ^2^University of Chinese Academy of Sciences, Beijing, China; ^3^Faculty of Geographical Science, Beijing Normal University, Beijing, China; ^4^Key Laboratory of Ecosystem Network Observation and Modeling, Institute of Geographic Sciences and Natural Resources Research, Chinese Academy of Sciences, Beijing, China

**Keywords:** angiosperm species, climate variability and seasonality, functional biogeography, leaf traits, phylogeny, plant growth form

## Abstract

Leaf traits play key roles in plant resource acquisition and ecosystem processes; however, whether the effects of environment and phylogeny on leaf traits differ between herbaceous and woody species remains unclear. To address this, in this study, we collected data for five key leaf traits from 1,819 angiosperm species across 530 sites in China. The leaf traits included specific leaf area, leaf dry matter content, leaf area, leaf N concentration, and leaf P concentration, all of which are closely related to trade-offs between resource uptake and leaf construction. We quantified the relative contributions of environment variables and phylogeny to leaf trait variation for all species, as well as for herbaceous and woody species separately. We found that environmental factors explained most of the variation (44.4–65.5%) in leaf traits (compared with 3.9–23.3% for phylogeny). Climate variability and seasonality variables, in particular, mean temperature of the warmest and coldest seasons of a year (MTWM/MTWQ and MTCM/MTCQ) and mean precipitation in the wettest and driest seasons of a year (MPWM/MPWQ and MPDM/MPDQ), were more important drivers of leaf trait variation than mean annual temperature (MAT) and mean annual precipitation (MAP). Furthermore, the responses of leaf traits to environment variables and phylogeny differed between herbaceous and woody species. Our study demonstrated the different effects of environment variables and phylogeny on leaf traits among different plant growth forms, which is expected to advance the understanding of plant adaptive strategies and trait evolution under different environmental conditions.

## Introduction

Leaf traits play multifaceted roles in plant resource acquisition (Wilson et al., 1999; Diaz et al., 2004), environmental adaptation (Reich and Oleksyn, 2004; Ordoñez et al., 2009), and ecosystem functions (Lavorel and Garnier, 2002; Ali et al., 2017). Leaf traits are highly variable across plant growth forms, phylogenetic groups, and environmental gradients, and reflect distinct plant responses to evolutionary history and environmental changes. Identifying the primary factors that influence leaf trait variation is helpful for understanding how plant assemblages drive community structures and ecosystem processes in response to global environmental change.

Climate and soil are important factors influencing interspecific variation in leaf traits and can affect both plant adaptive strategies and ecosystem functions (Wright et al., 2005a; Ordoñez et al., 2009; Maire et al., 2015). Plants in wetter, warmer, and more fertile conditions are characterized by greater specific leaf area (SLA) and leaf N concentration (LNC) but lower leaf dry matter content (LDMC), which can allow for faster resource uptake and lower construction costs by the leaves (Simpson et al., 2016; Delpiano et al., 2020). Regarding climate variables, some studies have suggested that climate variability and seasonality variables influence the variation in plant functional traits to a greater extent than annual temperature and precipitation averages (Moles et al., 2009; Shiono et al., 2015; Wright et al., 2017; Wang et al., 2019). Climate variability and seasonality variables generally refer to the temporal variability and fluctuations in temperature and precipitation, which can affect plant phenology, rate of photosynthesis, and the length of the growing season (Kikuzawa et al., 2013; Ernakovich et al., 2014). Similarly, phylogeny has also been suggested to have an important effect on leaf traits, some of which leaf traits [e.g., SLA, LNC, and leaf P concentration (LPC)] show significant phylogenetic conservatism among species (Kerkhoff et al., 2006; Stock and Verboom, 2012; Yang et al., 2016). Results concerning the relative importance of environment variables and phylogeny on leaf trait variation among different studies have been inconsistent. For instance, Li et al. (2021) suggested that LNC was more strongly influenced by environment variables than phylogeny in Southwest China, whereas He et al. (2010) reported that phylogeny and environment explained approximately equal proportions of the variation in LNC in grasslands in China. Determining the relative contributions of environment and phylogeny to leaf trait variation is important for advancing our understanding of plant adaptive strategies to environmental change.

Leaf traits have also been found to differ significantly among plant growth forms (Kerkhoff et al., 2006; Santiago and Wright, 2007). Relative to woody species, herbaceous species have greater SLA and LNC but lower LDMC, reflecting the pursual of a more resource-acquisitive strategy (Wright et al., 2005b; Rossatto and Franco, 2017). Diaz et al. (2016) also showed that herbaceous and woody plant groups represent two almost independent hotspots in the global spectrum of plant form and function. These observations suggest that the responses of leaf traits to environment variables and phylogeny may differ between herbaceous and woody species. In general, woody species have longer lifespans and higher phylogenetic conservatism, and thus tend to have more consistent functional similarity among lineages that share the same habitat when compared with herbaceous species (Lanfear et al., 2013; Flores et al., 2014). Additionally, some studies have demonstrated that the relationships between leaf traits and climate are dependent on plant growth form (Šímová et al., 2018; Gong et al., 2020). However, studies on the effects of environment variables and phylogeny on leaf traits have focused on woody species (Asner and Martin, 2016; Yang et al., 2016; de la Riva et al., 2017), herbaceous species (He et al., 2010), or the two in combination (Valverde-Barrantes et al., 2017; Yang et al., 2018), which may obscure leaf trait-environment relationships and different evolutionary processes among the two plant growth forms. Accordingly, an extensive comparative analysis of the effects of environment variables and phylogeny on leaf trait variation between herbaceous and woody species is critical for understanding plant responses to both external environments and evolutionary history (Cornwell et al., 2014).

Although several studies have reported on leaf trait-environment relationships or phylogenetic effects on leaf traits across different spatial scales (Ordoñez et al., 2009; Flores et al., 2014; Maire et al., 2015; Valverde-Barrantes et al., 2017; Šímová et al., 2018), none have investigated the combined effects of environment variables and phylogeny on leaf traits at a national level in China. Selective pressures can vary across spatial scales (Albert et al., 2010), and different traits may have different sensitivities to the same pressures (Messier et al., 2010). Importantly, China has large-scale climate gradients and biome types, ranging from tropical rainforests to deserts, that cover almost all global vegetation types (Fang et al., 2020). These represent ideal conditions for determining the relative contributions of environment variables and phylogeny to leaf traits for different plant growth forms.

In this study, we sought to determine the relative contributions of environment variables and phylogeny to leaf trait variation as well as identify how they differ between herbaceous and woody species in China. For this, we collected data for five key leaf traits from 1,819 terrestrial angiosperm species from 530 sampling sites in China, covering almost all major seed plant lineages and biome types. The leaf traits included SLA, LDMC, leaf area (LA), LNC, and LPC, all of which are closely associated with the leaf economics spectrum (LES), plant growth rate, and resource acquisition (Reich and Oleksyn, 2004; Wright et al., 2005a,^2017^).

## Materials and Methods

### Leaf Trait Data Collection

Leaf trait data (Table 1) were collected from three sources, namely, the TRY database (Kattge et al., 2011), the China Plant Traits Database^1^ (Wang et al., 2018), and published literature (a list of the data sources is presented in Supplementary Appendix A). The following criteria were used to ensure data quality and comparability: first, to minimize the effects of management disturbance, plant leaves were collected only from natural terrestrial ecosystems (excluding croplands, greenhouses, and laboratories); second, to reduce the confounding effects of ontogeny (Mason and Donovan, 2015), leaves were collected from healthy and mature plant individuals, while leaves in particular life stages (e.g., leaves from seedlings and expanding leaves) were excluded; third, leaves were sampled and measured according to the handbook for standardized measurement of plant functional traits (Perez-Harguindeguy et al., 2013); fourth, because plant ecological strategies may differ between native species and non-native species, leaves were collected only from the former (van Kleunen et al., 2010). Leaf samples were mostly collected during the peak of the growing season (June–August). In addition, when data for one species were collected from multiple sites, trait values for each site–species combination were recorded. When a species was reported at the same sampling site in more than one study, the mean of the trait values reported in these studies was used. Where leaf traits were presented only in figures, the trait values were extracted using GetData software (GetData Software Company, Kogarah, NSW, Australia). The geographical coordinates of the sampling sites were also recorded to obtain the corresponding environmental data.

**TABLE 1:** Description of the five leaf traits evaluated.

Leaf trait	Abbreviation	Unit	Functional role	References
Specific leaf area	SLA	m^2^ kg^–1^	Light capture, plant growth rate, and ecological strategy	Reich et al., 1991; Wright et al., 2004
Leaf dry matter content	LDMC	g g^–1^	Resistance to herbivory and drought and leaf litter decomposition	Pakeman et al., 2010; Yang et al., 2018
Leaf area	LA	cm^2^	Light interception and leaf energy balance related to photosynthesis, respiration, and evaporation	Wright et al., 2017; Yang et al., 2018
Leaf N concentration	LNC	mg g^–1^	Leaf metabolic activity, plant growth rate, and nutrient cycling	Reich and Oleksyn, 2004; Wright et al., 2004
Leaf P concentration	LPC	mg g^–1^	Leaf metabolic activity, plant growth rate, and nutrient cycling	Reich and Oleksyn, 2004; Wright et al., 2004

Species name and taxonomic nomenclature were standardized and corrected according to the *Flora of China*^2^ (Wu et al., 1994-2013) and an automated phylogeny assembly tool SoTree v2.0 in the DarwinTree platform^3^ (Meng et al., 2015). A total of 1,819 species from 147 families and 730 genera were collected in this study.

### Climate Data

Twenty climate variables were considered and divided into four groups, as follows (Supplementary Table 1):

1) *Light indicator group*: The variable included was mean annual sunlight (MASL).
2) *Temperature indicator group*: In addition to mean annual temperature (MAT), variables related to temperature variability and seasonality were also collected, that is, mean growing season temperature (GST); mean diurnal range (MDR); mean annual range of temperature (MTAR); mean temperature of the warmest month of a year (MTWM); mean temperature of the warmest quarter of a year (MTWQ); mean temperature of the coldest month of a year (MTCM); mean temperature of the coldest quarter of a year (MTCQ); and active accumulated temperature above 0°C (AA0).
3) *Precipitation indicator group*: In addition to mean annual precipitation (MAP), variables related to precipitation variability and seasonality were also collected, namely, mean growing season precipitation (GSP); mean annual range of precipitation (MPAR); mean precipitation of the wettest month of a year (MPWM); mean precipitation of the wettest quarter of a year (MPWQ); mean precipitation of the driest month of a year (MPDM); and mean precipitation of the driest quarter of a year (MPDQ).
4) *Aridity indicator group*: Variables included potential evapotranspiration (PET), actual evapotranspiration (AET), and aridity index (AI, the ratio between MAP and AET).

Excluding AA0, AET, AI, and PET, the above-mentioned climate variables were collected between 1990 and 2015 by 824 national meteorological stations and interpolated to a 1 km × 1 km grid with elevation as a covariate using the interpolation software ANUSPLIN (Hancock and Hutchinson, 2006). Values for the AA0 were obtained from the Data Center for Resources and Environmental Sciences, Chinese Academy of Sciences, with a spatial resolution of 500 m.^4^ AET-, AI- and PET-related data were extracted from the CGIAR-CSI GeoPortal dataset and were collected between 1950 and 2000 (Trabucco and Zomer, 2009).^5^ All climate variables were resampled to 1 km. Notably, the MAT and MAP from the original literature agreed well with the values in our interpolated dataset (all *R*^2^ values were >0.95, *p* < 0.05). Consequently, the interpolated data were used in subsequent analyses.

### Soil Data

Data for a total of 17 soil variables were collected and divided into two groups, as follows (Supplementary Table 1):

1) *Physical indicator group*: Variables included the percentage of sand fraction (SAND), percentage of silt fraction (SILT), percentage of clay fraction (CLAY), and bulk density (BD).
2) *Chemical indicator group*: Variables included pH, soil organic matter (SOM), soil organic carbon (SOC), soil total N (STN), soil total P (STP), soil total K (STK), clay cation exchange capacity (CCEC), soil cation exchange capacity (SCEC), base saturation (BS), total exchangeable bases (TEB), calcium carbonate content (CaCO_3_), exchangeable sodium percentage (ESP), and electrical conductivity (EC).

Data for SOM, STN, STP, and STK were extracted from the National Earth System Science Data Sharing Infrastructure, National Science & Technology Infrastructure of China,^6^ and the spatial resolution was 1 km. The remaining variables were obtained from the Harmonized World Soil Database v1.2^7^ (FAO et al., 2012).

### Data Analysis

Before analysis, leaf trait data were log_10_-transformed to meet the approximate normality and homogeneity of the residuals. Climate and soil variables were subjected to Yeo-Johnson transformation to reduce skewness and allow for the normalized transformation of non-positive values using the “*bestNormalize*” R package (Peterson and Cavanaugh, 2019; Supplementary Table 2).

A phylogenetic tree was constructed based on the published phylogenetic tree of Chinese vascular plants developed by Chen et al. (2016) and updated by Hu et al. (2020). As the largest and most up-to-date species-level phylogeny of Chinese vascular plants, this phylogeny was based on sequences of chloroplast and mitochondrial genes and covered 95.7% of genera native to China. This phylogenetic tree in this study was constructed via SoTree v2.0 tool in the DarwinTree platform (see text footnote 3). All 1,819 species were included in this phylogenetic tree (Supplementary Figure 1). The phylogenetic tree was visualized in Interactive Tree of Life (iTOL)^8^ (Letunic and Bork, 2019). Given that the incorporation of phylogenetic information in further statistical analysis required fully resolved phylogenetic trees, polytomies within the phylogeny were resolved using the “multi2di” function in the “*ape*” R package (Paradis and Schliep, 2019).

Pagel’s λ (Pagel, 1999) was calculated to examine the phylogenetic signals of the leaf traits. A λ value close to zero indicates a weak phylogenetic signal for a given trait, while a value close to one suggests a strong phylogenetic signal (Freckleton et al., 2002). This analysis was performed using the “*phytools*” R package (Revell, 2012).

To consider the phylogenetic effect among species, a phylogenetic linear mixed model (PLMM) was used to examine the relationship between leaf traits and climate and soil variables using residual maximum likelihood (REML) estimation. This approach offers a flexible framework that includes the phylogenetic relationships among species to reduce the variance in the estimated regression or correlation coefficients (Rohlf, 2006; Chamberlain et al., 2012) and overcomes the limitation of phylogenetic generalized least squares that allows a specific species to correspond to only one observation. In the model, each climate and soil variable was treated as a fixed effect, species was treated as a random effect and the distance matrix among species was treated as a covariance. The sums of squares explained by fixed effects and their significance were estimated using the “r.squaredGLMM” function and “Anova” function in the “*lme4qtl*” R package, respectively (Ziyatdinov et al., 2018).

The PLMM was further used to partition the individual contributions of environment variables and phylogeny to the variation in leaf traits for all species as well as herbaceous and woody plant groups, respectively. The phylogenetic effect was defined as a hierarchically nested structure “clade/order/family.” The phylogenetic clades were classified into the following seven groups according to Angiosperm Phylogeny Group IV (APG IV) classification (APG, 2016): basal angiosperms, chloranthales, magnoliids, monocots, basal eudicots, superrosids, and superasterids (Hu et al., 2020). Basal eudicots indicated unranked eudicot species, i.e., species that were not included in the superrosids and superasterids clades. The overall random term within the variance components model was “environment + [clade/order/family],” and no fixed factor was defined. The variation in leaf traits due to climate and soil was assigned to the “environment” component in the regression models (Watanabe et al., 2007; Zhao et al., 2016). Given the large number of environmental factors involved and their collinearity (Supplementary Figures 2, 3), a principal component analysis (PCA) was performed to reduce the number of dimensions of less important environmental factors. Thus, the first three principal components accounted for 72.2% of the total variance and were used in the models to represent the “environment” component (Supplementary Table 3). In addition, the interactive effects of environment variables and phylogenetic structure were labeled as “overlap.”

All above-mentioned analyses were performed using R 3.4.3 (R Core Team, 2017). The significance of differences in leaf traits among plant growth forms and phylogenetic clades were examined using one-way ANOVA with Tukey’s *post hoc* tests in SPSS software v17 (SPSS Inc., Chicago, IL, United States).

## Results

### Leaf Trait Variation

**FIGURE 1:**
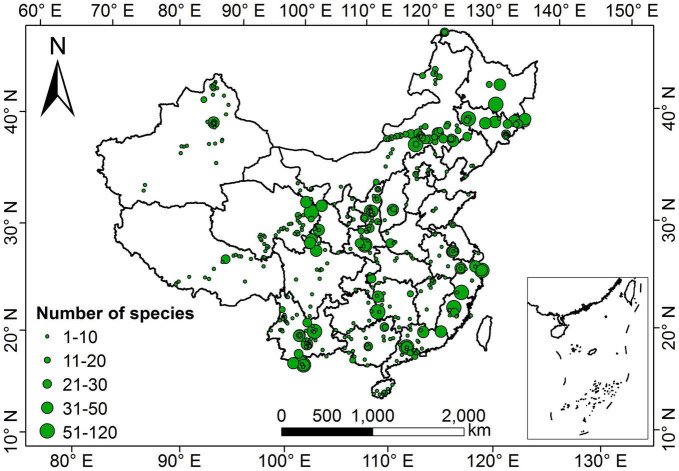
Geographical distribution of sampling sites in China. The size of the green dots on the map indicates the relative number of species measurements.

**FIGURE 2:**
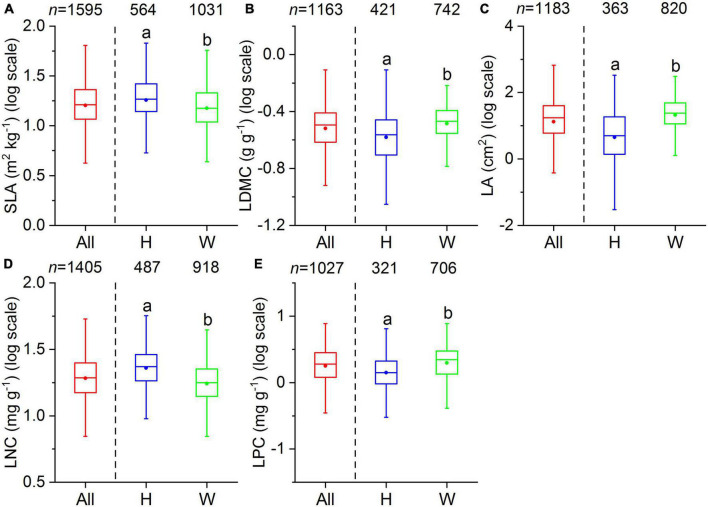
Boxplots of leaf traits for all species (All), herbaceous species (H), and woody species (W). The number above each box indicates the total number of species per group. The bottom and top of the boxplots indicate the first and third quartile, the two whiskers correspond to the 1.5 times of the outliers, and the solid dots within the boxes are the mean values. Different lowercase letters indicate significant differences between herbaceous and woody species (*p* < 0.05). **(A)** SLA, specific leaf area; **(B)** LDMC, leaf dry matter content; **(C)** LA, leaf area; **(D)** LNC, leaf N concentration; **(E)** LPC, leaf P concentration.

Our dataset comprised 1,819 terrestrial angiosperm species from 530 sampling sites (Figure 1). The five leaf traits showed large variability across species (Figure 2). LA showed the largest variation (more than 1000-fold), varying from 0.003 to 1085.61 cm^2^. SLA varied by more than 90-fold among species, ranging from 0.83 to 75.14 m^2^ kg^–1^. For leaf nutrient traits, differences of up to 60-fold for LNC (range: 0.09 to 60.10 mg g^–1^) and 70-fold for LPC (range: 0.11 to 7.74 mg g^–1^) were detected. Significant differences were observed between herbaceous and woody plant groups (*p* < 0.05). Herbaceous species had smaller LA, greater SLA, and higher LNC, and lower LDMC and LPC compared with woody species.

### Phylogenetic Signals of the Leaf Traits

The five leaf traits displayed significant phylogenetic signals for all species as well as herbaceous and woody species (Table 2). Generally, the phylogenetic signals of LNC and LPC were relatively lower than SLA, LDMC, and LA. Also, the five leaf traits showed significant differences among different phylogenetic clades (*p* < 0.05, Table 3). The early-diverged clades (e.g., magnoliids) had larger leaves, smaller SLA, and lower LNC, but higher LDMC than the late-diverged clades (e.g., superasterids and superrosids).

**TABLE 2:** Phylogenetic signals of the leaf traits evaluated.

	All species		Herbaceous species		Woody species	
	Pagel’s λ	*p*	Pagel’s λ	*p*	Pagel’s λ	*p*
SLA	0.680	<0.001	0.660	<0.001	0.752	<0.001
LDMC	0.788	<0.001	0.770	<0.001	0.668	<0.001
LA	0.839	<0.001	0.656	<0.001	0.781	<0.001
LNC	0.646	<0.001	0.449	<0.001	0.616	<0.001
LPC	0.610	<0.001	0.353	0.03	0.556	<0.001

*The leaf trait values are log_10_-transformed.*

*SLA, specific leaf area; LDMC, leaf dry matter content; LA, leaf area; LNC, leaf N concentration; LPC, leaf P concentration.*

**TABLE 3:** Differences in the five leaf traits among phylogenetic clades.

Phylogenetic clade	SLA	LDMC	LA	LNC	LPC
Basal angiosperms*	22.37 ± 11.46	0.23 ± 0.04	63.81 ± 48.42	13.45 ± 4.91	1.95 ± 1.48
Chloranthales*	44.14 ± 5.61	0.14 ± 0.05	9.48 ± 6.21	29.77	4.80
Magnoliids	14.82 ± 7.46a	0.38 ± 0.10a	76.60 ± 152.20a	17.46 ± 5.44a	2.44 ± 1.29a
Monocots	19.94 ± 10.06bc	0.33 ± 0.13b	34.32 ± 110.63b	21.44 ± 7.12b	1.62 ± 0.99b
Basal eudicots	21.70 ± 12.57b	0.29 ± 0.08c	33.38 ± 45.41b	23.05 ± 8.86b	1.65 ± 0.86bc
Superrosids	18.83 ± 11.21c	0.35 ± 0.10ab	38.72 ± 60.43b	21.46 ± 8.01b	2.27 ± 1.15a
Superasterids	18.53 ± 10.16bc	0.28 ± 0.10c	30.57 ± 48.04b	20.17 ± 9.23b	2.06 ± 1.16ac
*p-*Value	<0.001	<0.001	<0.001	<0.001	<0.001

*Letters indicate significant differences among phylogenetic clades (p < 0.05).*

*Basal angiosperms and chloranthales is not included in ANOVA analyses due to few species (n < 5).

*SLA, specific leaf area; LDMC, leaf dry matter content; LA, leaf area; LNC, leaf N concentration; LPC, leaf P concentration.*

### Relationships Between Leaf Traits and Climate and Soil Variables

**FIGURE 3:**
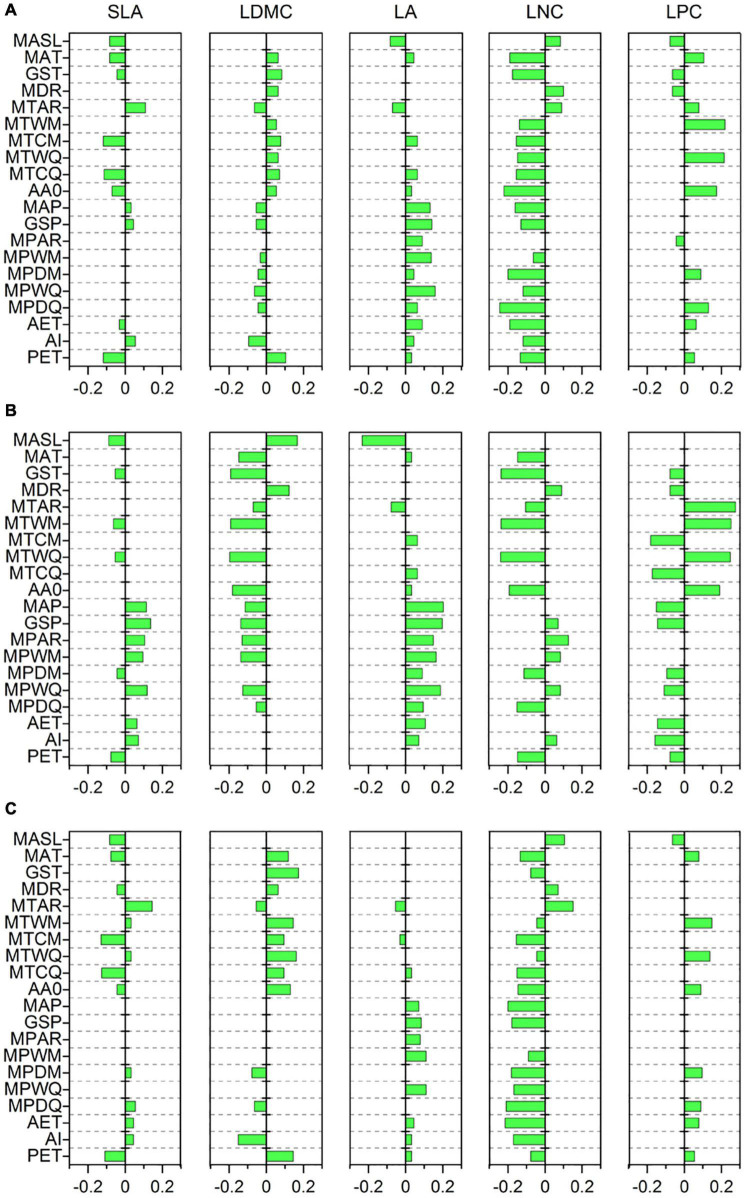
Correlations of leaf traits with climate variables based on a phylogenetic linear mixed model (PLMM). Significant correlations between leaf traits and climate variables are shown (*p* < 0.05). Positive values represent positive correlations between leaf traits and climate variables, while negative values represent negative correlations between them. **(A)** All species; **(B)** herbaceous species; **(C)** woody species. SLA, specific leaf area; LDMC, leaf dry matter content; LA, leaf area; LNC, leaf N concentration; LPC, leaf P concentration; MASL, mean annual sunlight; MAT, mean annual temperature; GST, mean growing season temperature; MDR, mean diurnal range; MTAR, mean annual range of temperature; MTWM, mean temperature of the warmest month of a year; MTCM, mean temperature of the coldest month of a year; MTWQ, mean temperature of the warmest quarter of a year; MTCQ, mean temperature of the coldest quarter of a year; AA0, active accumulated temperature above 0°C; MAP, mean annual precipitation; GSP, mean growing season precipitation; MPAR, mean annual range of precipitation; MPWM, mean precipitation of the wettest month of a year; MPDM, mean precipitation of the driest month of a year; MPWQ, mean precipitation of the wettest quarter of a year; MPDQ, mean precipitation of the driest quarter of a year; AET, actual evapotranspiration; AI, aridity index; PET, potential evapotranspiration.

**FIGURE 4:**
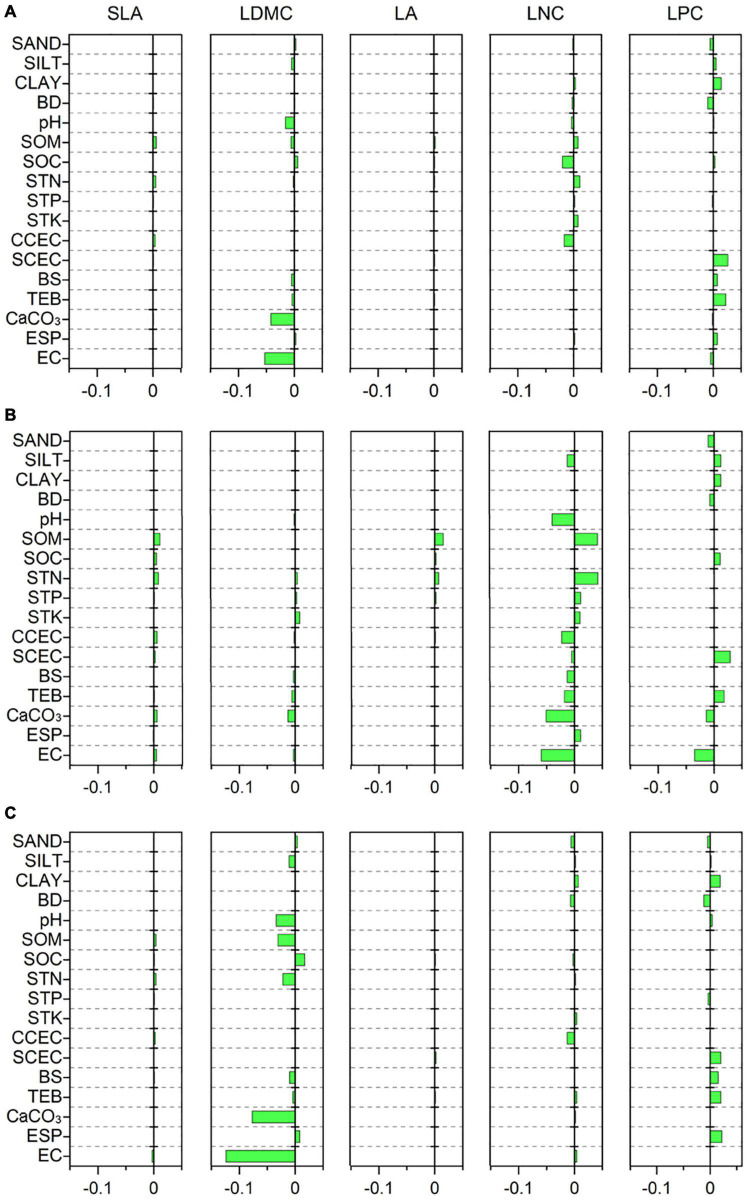
Correlations of leaf traits with soil variables based on a phylogenetic linear mixed model (PLMM). Significant correlations between leaf traits and soil variables are shown (*p* < 0.05). Positive values represent positive correlations between leaf traits and soil variables, while negative values represent negative correlations between them. **(A)** All species; **(B)** herbaceous species; **(C)** woody species. SLA, specific leaf area; LDMC, leaf dry matter content; LA, leaf area; LNC, leaf N concentration; LPC, leaf P concentration; SAND, percentage of sand fraction; SILT, percentage of silt fraction; CLAY, percentage of clay fraction; BD, bulk density; SOM, soil organic matter; SOC, soil organic carbon; STN, soil total N; STP, soil total P; STK, soil total K; CCEC, clay cation exchange capacity; SCEC, soil cation exchange capacity; BS, base saturation; TEB, total exchangeable bases; CaCO_3_, calcium carbonate content; ESP, exchangeable sodium percentage; EC, electrical conductivity.

Specific leaf area was strongly related to MTCM, MTCQ, and PET (*R* = −0.118, −0.114, and −0.118, respectively, all *p* < 0.001) (Figure 3A). The relationships between LDMC and most climate variables were significant but weak (|*R*| < 0.11). LA showed strong associations with precipitation indices and had the closest relationships with MPWM and MPWQ (*R* = 0.138 and 0.158, respectively, both *p* < 0.001). LNC was significantly related to most climate variables and showed the strongest correlations with MPDM and MPDQ (*R* = −0.200 and −0.245, respectively, both *p* < 0.001). LPC was most strongly linked to MTWM and MTWQ (*R* = 0.219 and 0.214, respectively, both *p* < 0.001). Most soil variables, especially those associated with soil fertility, showed weak or insignificant associations with leaf traits (Figure 4A); however, LDMC was significantly and negatively correlated with pH, CaCO_3_, and EC (*R* = −0.126, −0.205 and −0.230, respectively, both *p* < 0.001).

The relationships between leaf traits and environment variables showed large variation between herbaceous and woody species. SLA of woody species was more strongly related to MTCM and MTCQ (*R* = −0.130 and −0.126, respectively, both *p* < 0.001) compared with that from herbaceous species (Figures 3B,C). LDMC and LPC were closely related to MTWM and MTWQ for both herbaceous and woody species. LA of herbaceous species was more strongly influenced by precipitation indices than temperature indices, especially MAP, GSP, MPWM, and MPWQ (both *R* > 0.150, both *p* < 0.001). The LNC of herbaceous species displayed the closest links to MTWM and MTWQ (*R* = −0.237 and −0.239, respectively, both *p* < 0.001), whereas that from woody species was more strongly related to precipitation indices than temperature indices, especially MPDM and MPDQ (*R* = −0.182 and −0.210, respectively, both *p* < 0.001). Among the soil fertility variables, the LNC of herbaceous species showed strong associations with SOM and STN (*R* = 0.202 and 0.205, respectively, both *p* < 0.001) (Figure 4B). LDMC for the woody species and LNC for the herbaceous species were negatively correlated with CaCO_3_ and EC (both *R* < −0.20, all *p* < 0.001) (Figures 4B,C).

### The Relative Contributions of Environment Variables and Phylogeny to Leaf Traits

**FIGURE 5:**
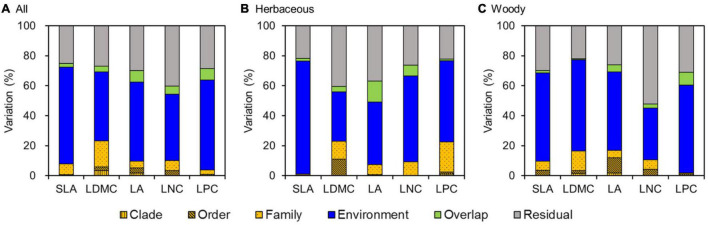
Variance partitioning for leaf traits using a phylogenetic linear mixed model (PLMM) for **(A)** all species, **(B)** herbaceous species, and **(C)** woody species. SLA, specific leaf area; LDMC, leaf dry matter content; LA, leaf area; LNC, leaf N concentration; LPC, leaf P concentration.

Analysis of the PLMM results showed that environment variables explained most of the variation in leaf traits, accounting for 44.4–65.5% of the total variation (Figure 5A). Meanwhile, phylogeny had only weak effects on leaf traits, explaining 3.9–23.3% of the total variation. The effects of phylogeny were mainly observed at the family level, especially for LDMC (17.4%), LNC (10.1%), and SLA (7.2%).

Similarly, environment variables were primary sources of variation in leaf traits for both herbaceous and woody species, accounting for 32.9–75.3 and 34.4–60.9% of the total variation for herbaceous and woody species, respectively (Figures 5B,C). Phylogeny also had weak effects on leaf traits for both herbaceous and woody species (1.1–22.9 vs 1.8–16.8%, respectively). In addition, the effects of environment variables and phylogeny on herbaceous and woody species differed among leaf traits. SLA, LA, and LNC for herbaceous species were less influenced by phylogenetic structure compared with woody species. However, LDMC and LPC of woody species were more influenced by environment variables than those of herbaceous species.

## Discussion

### The Effects of Environment Variables on Leaf Traits

Identifying the relationships between leaf traits and the environment variables is helpful for better understanding the impacts of environmental filtering on trait value selection and paleoclimate reconstruction (Peppe et al., 2011; Van Bodegom et al., 2012). Our results showed that environment variables explained the largest proportion of the variation in leaf traits, which was inconsistent with the results of a study by Yang et al. (2018), where the authors reported that environment variables exerted relatively weak effects on SLA and LDMC. A possible explanation for this discrepancy may be that Yang et al. (2018) sampled only a limited number of sites at each biome in eastern and southwestern China, which may have influenced the values for responses of leaf traits to environmental conditions. In particular, our study revealed that climate variability and seasonality variables were more important than MAT and MAP as drivers of leaf trait variation. This result highlighted the importance of climate variability and seasonality variables in regulating the variation in plant functional traits (Moles et al., 2009; Shiono et al., 2015; Wright et al., 2017). For instance, precipitation in the wettest season (i.e., MPWM and MPWQ) was the factor that most (positively) influenced LA variation. A smaller LA could be an adaptative strategy to decrease water loss *via* reducing the surface area for transpiration under dry environmental conditions (Picotte et al., 2007; Du et al., 2019). In the present study, temperature in the warmest season (i.e., MTWM and MTWQ) accounted for the greatest proportion of LNC variation among herbaceous species, and species growing in areas with lower MTWM/MTWQ tended to have higher LNC. This may be related to the fact that species with higher LNC generally have higher rates of photosynthetic C fixation, which would thus offset the lower rates of biochemical reactions observed in cold climates (McGroddy et al., 2004; Reich and Oleksyn, 2004). However, for woody species, precipitation in the driest season (i.e., MPDM and MPDQ) was an important factor for the variation in LNC, and negative relationships were found between the two. A higher LNC can enhance the exploitation of light availability, thereby maximizing the photosynthetic rate under conditions of low precipitation (Cunningham et al., 1999; Wright et al., 2003; Santiago et al., 2004). Overall, these findings identified climate variability and seasonality variables as critical links between resource conditions and plant adaptive strategies, thereby contributing our understanding of leaf trait-climate relationships under future global changes.

In contrast to previously reported results (Ordoñez et al., 2009; Pakeman, 2013; Gong et al., 2020), we found only weak correlations between leaf traits and soil fertility variables. The weakness of these correlations may be partially related to the complexity of soil fertility-mediated regulation on leaf traits, such as the interactive effects among soil nutrients, soil-climate interactions, or differences in the type and magnitude of nutrient limitation (Greenwood et al., 2004; Fujita et al., 2013; Simpson et al., 2016). However, we found strong and negative relationships between LDMC and pH or CaCO_3_. This indicated that plants tended to invest more in dry matter in nutrient-poor environments, which can enable plants to have high construction costs and longer lifespans (Hodgson et al., 2011; Jager et al., 2015; Maire et al., 2015). Strong and negative relationships were also observed between LDMC and EC, in contrast to the positive or no correlations reported by other studies (Wang et al., 2015; De Battisti et al., 2020). This discrepancy may be attributable to differences in species composition between our and previous studies that may have resulted in heterogeneous responses to soil salinity. For instance, our study comprised a large number of species, whereas other studies examined only one species or relatively few halophyte species (Wang et al., 2015; De Battisti et al., 2020).

In addition, we found that environment variables explained most of variation in leaf traits for both herbaceous and woody species. However, the strength of environmental effects on herbaceous and woody species varied among leaf traits. For example, LDMC, LA, and LPC for woody species were more influenced by environment variables compared with herbaceous species, which is consistent with a previous study reporting that LPC was more environmentally constrained than phylogenetic effect in shrubland biomes (Yang et al., 2016). This reflected that woody and herbaceous species have different ecological strategies to cope with changing environments, especially fluctuating and seasonally inhospitable climates (Zanne et al., 2014; Qian et al., 2017). Compared with herbaceous plants, woody plants generally with larger plant sizes and higher investment in tissue construction and maintenance (e.g., higher LDMC) expose in climate, which can facilitate adaptation to unfavorable environments (Ricklefs and Latham, 1992; Wang et al., 2020; Zhou et al., 2020). Further studies that explore which leaf traits strongly respond to climate conditions for different plant growth forms could improve our understanding of the effects of climate changes on ecosystem functions.

### The Effects of Phylogeny on Leaf Traits

Our results demonstrated that phylogeny had a weak effect on leaf trait variation, which was in contrast to recent studies that have reported phylogeny as the most important contributor to the variance in leaf traits (Valverde-Barrantes et al., 2017; Yang et al., 2018). We attributed this inconsistency to the different evolutionary processes associated with the different taxa and phylogenetic scales studied (Figueroa and Smith, 2020; Valverde-Barrantes et al., 2020). The weak effects of phylogeny were indicative of evolutionary lability during seed plant diversification, resulting in considerable trait distinctiveness across lineages (Cornwell et al., 2014; Amaral et al., 2021). Furthermore, most of the phylogenetic effects on leaf traits were observed at the family level, which is consistent with the findings of previous studies (Yang et al., 2018; Zhang et al., 2019). This result is also in line with evidence indicating that plant functional traits are conserved at the family level (Kerkhoff et al., 2006; Qian and Zhang, 2016), suggesting that different patterns of geographic distribution of various families reflect their adaptations to specific geographical and climatic contexts (Cornwell et al., 2014).

We found that phylogenetic effects on herbaceous and woody species also differed among leaf traits. SLA, LA, and LNC for herbaceous species were less influenced by phylogeny compared with woody species. This result is consistent with the study of Flores et al. (2014) who found that leaf mass per area of herbaceous species showed lower phylogenetic conservatism than that of woody species, and also partly supported previous studies suggesting that herbaceous plants have higher evolutionary rate, phylogenetic turnover and lower phylogenetic conservatism compared with woody plants (Smith and Donoghue, 2008; Smith and Beaulieu, 2009; Hawkins et al., 2011). However, LDMC and LPC for herbaceous were more influenced by phylogenetic effects compared with woody species in this present study, which supported the above-mentioned results in which these traits were more environmentally constrained. The different responses of leaf traits to phylogeny between herbaceous and woody species may result from differences in evolutionary processes (e.g., evolutionary rate and stabilizing selection) (Flores et al., 2014; Zhou et al., 2018; Wang et al., 2020).

### The Interactive Effects of Environment Variables and Phylogeny on Leaf Traits

In the present study, we detected interactive effects of environment variables and phylogeny on leaf traits, indicating that phylogenetic structure across lineages is mediated by environmental filtering (Crisp et al., 2009; Gagnon et al., 2019). For instance, Proteaceae with lower LNC for a given SLA were found to be more frequent in infertile soils, suggesting that they are associated with a slow return position on the LES (Cornwell et al., 2014; Delgado et al., 2018). Major evolutionary lineages that were restricted to specific environmental conditions may reflect their preferred environmental regimes (Sanchez-Martinez et al., 2020). Therefore, further studies on distinctive plant lineage-trait combinations under different environmental contexts are helpful for elucidating the shifts in plant trait syndromes under changing environmental conditions.

### Uncertainties and Research Directions

In our study, we used a large leaf trait dataset to elucidate the relative importance of environment variables and phylogeny on leaf traits between herbaceous and woody species in China; however, some uncertainties remain. First, the limited number of species evaluated from habitats in northwestern China, i.e., deserts and alpine tundra, may have biased the interpretation of leaf trait variation and their relationships with environmental factors. Future studies should include a greater number of species from this area of China to test the generality of our results. Second, we used a species-level phylogenetic tree of Chinese vascular plants that was based on a variety of molecular sequences (Chen et al., 2016; Hu et al., 2020). Some studies have demonstrated that phylogenies representing different levels of phylogenetic resolution (i.e., family, genus, and species) may result in some bias in quantification of phylogenetic structure (Molina-Venegas and Roquet, 2014; Qian and Jin, 2021). Further studies in developing phylogenies well resolved at the genus or species level with more species and gene markers will help for better understanding the geographical patterns of plant traits at the large scale (Janssens et al., 2020). Third, our measures of soil variables were represented by the large-scale estimations of soil properties, which may not reflect the high heterogeneity of soil properties at multiple spatial scales and may introduce some uncertainties regarding the correlations between leaf traits and soil variables. Finally, we did not consider the interactive effects of climate and soil on leaf trait variation. The geographical patterns of leaf traits in a given climate may depend on the level of soil fertility (Ordoñez et al., 2009; Simpson et al., 2016). Future studies that determine the effects of climate-soil interactions on leaf trait variation should improve our understanding of plant community responses to environmental change.

## Conclusion

Our study provided a comprehensive analysis of how environment variables and phylogeny contribute to the variation in key leaf traits between herbaceous and woody plant species in China. Environment variables explained most of the variation in leaf traits, whereas phylogeny had only a weak effect. Our study highlighted the importance of climate variability and seasonality variables in determining leaf trait variation, providing important information regarding ecological adaptations used by plants to cope with seasonal and fluctuating climatic conditions. Furthermore, the responses of leaf traits to environment variables and phylogeny were different between herbaceous and woody species, suggesting that shifts in plant growth form are important factors in predicting changes in plant traits and ecosystem functions under future climate change.

## Data Availability Statement

The raw data supporting the conclusions of this article will be made available by the authors, without undue reservation.

## Author Contributions

NA conceived the ideas and collected the data. NA and NL led the writing of the manuscript. All authors contributed critically to the drafts and gave the final approval for publication.

## Conflict of Interest

The authors declare that the research was conducted in the absence of any commercial or financial relationships that could be construed as a potential conflict of interest.

## Publisher’s Note

All claims expressed in this article are solely those of the authors and do not necessarily represent those of their affiliated organizations, or those of the publisher, the editors and the reviewers. Any product that may be evaluated in this article, or claim that may be made by its manufacturer, is not guaranteed or endorsed by the publisher.

## Footnotes

1
http://onlinelibrary.wiley.com/doi/10.1002/ecy.2091/suppinfo

2
http://www.iplant.cn/frps

3
http://www.darwintree.cn/flora-sotree-v2/index.shtml

4
http://www.resdc.cn

5
https://srtm.csi.cgiar.org/

6
http://www.geodata.cn

7
http://webarchive.iiasa.ac.at/Research/LUC/External-World-soil-database/HTML/

8
https://itol.embl.de/
