# Supplementary material for: Distinct Responses of Leaf Traits to Environment and Phylogeny Between Herbaceous and Woody Angiosperm Species in China
Source: Front Plant Sci. 2021 Dec 7;12:799401. doi: 10.3389/fpls.2021.799401 (PMC8688848; doi:10.3389/fpls.2021.799401)
Supplement: Supplementary file 1 [file Data_Sheet_1.docx]

**Supplementary data**

**Supplementary Table 1** Details on climate and soil variables and their biogeographic representation in this study.

| Variables | Units | Description | Database |
| --- | --- | --- | --- |
| *Light indicator* |  |  |  |
| MASL | h yr^-1^ | Mean Annual Sun Light | Calculated |
| *Temperature indicator* |  |  |  |
| MAT | ℃ | Mean Annual Temperature | Calculated |
| GST | ℃ | Mean Growing Season Temperature | Calculated |
| MTWM | ℃ | Mean Temperature of the Warmest Month | Calculated |
| MTCM | ℃ | Mean Temperature of the Coldest Month | Calculated |
| MDR | ℃ | Mean Diurnal Range (daily max temp-daily min temp) | Calculated |
| MTAR | ℃ | Mean Temperature of Annual Range (MTWM-MTCM) | Calculated |
| MTWQ | ℃ | Mean Temperature of the Warmest Quarter | Calculated |
| MTCQ | ℃ | Mean Temperature of the Coldest Quarter | Calculated |
| AA0 | ℃ | Active Accumulated Temperature Above 0℃ | RESDC |
| *Precipitation indicator* |  |  |  |
| MAP | mm | Mean Annual Precipitation | Calculated |
| GSP | mm | Mean Growing Season Precipitation | Calculated |
| MWP | mm | Mean Precipitation of the Wettest Month | Calculated |
| MDP | mm | Mean Precipitation of the Driest Month | Calculated |
| MPAR | mm | Mean Precipitation of Annual Range (MPWM-MPDM) | Calculated |
| MPWQ | mm | Mean Precipitation of the Wettest Quarter | Calculated |
| MPDQ | mm | Mean Precipitation of the Driest Quarter | Calculated |
| *Aridity indicator* |  |  |  |
| AI | / | Aridity Index | CGIAR-CSI |
| PET | / | Potential Evapotranspiration | CGIAR-CSI |
| AET | mm | Actual Evapotranspiration | CGIAR-CSI |
| *Soil physical indicator* |  |  |  |
| SAND | % weight | Percentage of Sand Fraction | HWSD |
| SILT | % weight | Percentage of Silt Fraction | HWSD |
| CLAY | % weight | Percentage of Clay Fraction | HWSD |
| BD | kg dm^-3^ | Bulk Density | HWSD |
| *Soil chemical indicator* |  |  |  |
| SOM | g kg^-1^ | Soil Organic Matter | http://www.geodata.cn |
| STN | g kg^-1^ | Soil Total Nitrogen | http://www.geodata.cn |
| STP | g kg^-1^ | Soil Total Phosphorus | http://www.geodata.cn |
| STK | g kg^-1^ | Soil Total Kalium | http://www.geodata.cn |
| pH | -log (H+) | / | HWSD |
| SOC | % weight | Topsoil Organic Carbon | HWSD |
| CCEC | cmol kg^-1^ | Clay Cation Exchange Capacity | HWSD |
| SCEC | cmol kg^-1^ | Soil Cation Exchange Capacity | HWSD |
| BS | % | Base Saturation | HWSD |
| TEB | cmol kg^-1^ | Total Exchangeable Bases | HWSD |
| CaCO_3_ | % weight | Calcium Carbonate Content | HWSD |
| ESP | % | Exchangeable Sodium | HWSD |
| EC | dS m^-1^ | Electrical Conductivity | HWSD |

For the 530 sites we obtained the variables from the following climate and soil international databases: Data Center for Resources and Environmental Sciences, Chinese Academy of Sciences (RESDC); Harmonised World Soil Database (FAO et al., 2012); National Earth System Science Data Sharing Infrastructure, National Science & Technology Infrastructure of China (<http://www.geodata.cn>). Global Aridity Index (Global-Aridity) and Global Potential Evapo-Transpiration (Global-PET) Geospatial Database. CGIAR Consortium for Spatial Information. Published online, available from the CGIAR-CSI GeoPortal at: <http://www.csi.cgiar.org>.

**Supplementary Table 2** Value of lambda for the environmental parameter transformation.

| Climate variables | Lambda |  | Soil variables | Lambda |
| --- | --- | --- | --- | --- |
| MASL | 1.408 |  | SAND | 0.477 |
| MAT | 0.846 |  | SILT | 1.071 |
| GST | 1.521 |  | CLAY | 0.332 |
| MDR | 1.207 |  | BD | 2.331 |
| MTAR | 0.496 |  | pH | 1.436 |
| MTWM | 2.105 |  | SOM | 0.195 |
| MTCM | 1.004 |  | SOC | -1.525 |
| MTWQ | 1.970 |  | STN | -0.270 |
| MTCQ | 0.986 |  | STP | -1.708 |
| AA0 | 0.667 |  | STK | 2.000 |
| MAP | 0.482 |  | CCEC | 0.457 |
| GSP | 0.536 |  | SCEC | -0.048 |
| MPAR | 0.682 |  | BS | 1.764 |
| MPWM | 0.704 |  | TEB | 0.181 |
| MPDM | -0.109 |  | CaCO_3_ | -2.494 |
| MPWQ | 0.670 |  | ESP | -0.101 |
| MPDQ | 0.080 |  | EC | -5.000 |
| AET | 1.195 |  |  |  |
| AI | 0.692 |  |  |  |
| PET | 1.287 |  |  |  |

See Table S1 for abbreviations.

**Supplementary Table 3** Principal component analyses of climate and soil variables.

|  | PC1 | PC2 | PC3 |
| --- | --- | --- | --- |
| Standard deviation | 3.42 | 2.05 | 1.45 |
| Proportion of variance (%) | 46.9 | 16.9 | 8.4 |
| SSD | 0.25 | 0.13 |  |
| MAT | -0.26 | 0.19 |  |
| GST | -0.22 | 0.28 |  |
| MDR | 0.24 |  |  |
| MTAR | 0.21 | 0.13 |  |
| MTCQ | -0.27 |  |  |
| MTWQ | -0.20 | 0.30 |  |
| MAP | -0.27 | -0.15 |  |
| GSP | -0.27 | -0.16 |  |
| MPAR | -0.21 | -0.18 |  |
| MPWQ | -0.26 | -0.17 |  |
| MPDQ | -0.26 |  |  |
| AA0 | -0.25 | 0.21 |  |
| AI | -0.23 | -0.22 |  |
| PET | -0.22 | 0.22 |  |
| AET | -0.28 |  |  |
| SOM |  | -0.44 |  |
| STN |  | -0.44 |  |
| STP |  | -0.26 |  |
| STK | 0.17 |  |  |
| CLAY |  | -0.11 | 0.64 |
| BD |  |  | -0.64 |
| SOC |  |  | 0.18 |
| pH |  |  |  |
| CCEC |  |  | -0.31 |

Note that MTWM, MTCM, MPWM, and MPDM as being highly redundant with MTWQ, MTCQ, MPWQ, and MPDQ, respectively (Figure S2). Soil variables in the table are selected based on the significant relationships with leaf traits. See Table S1 for abbreviations.


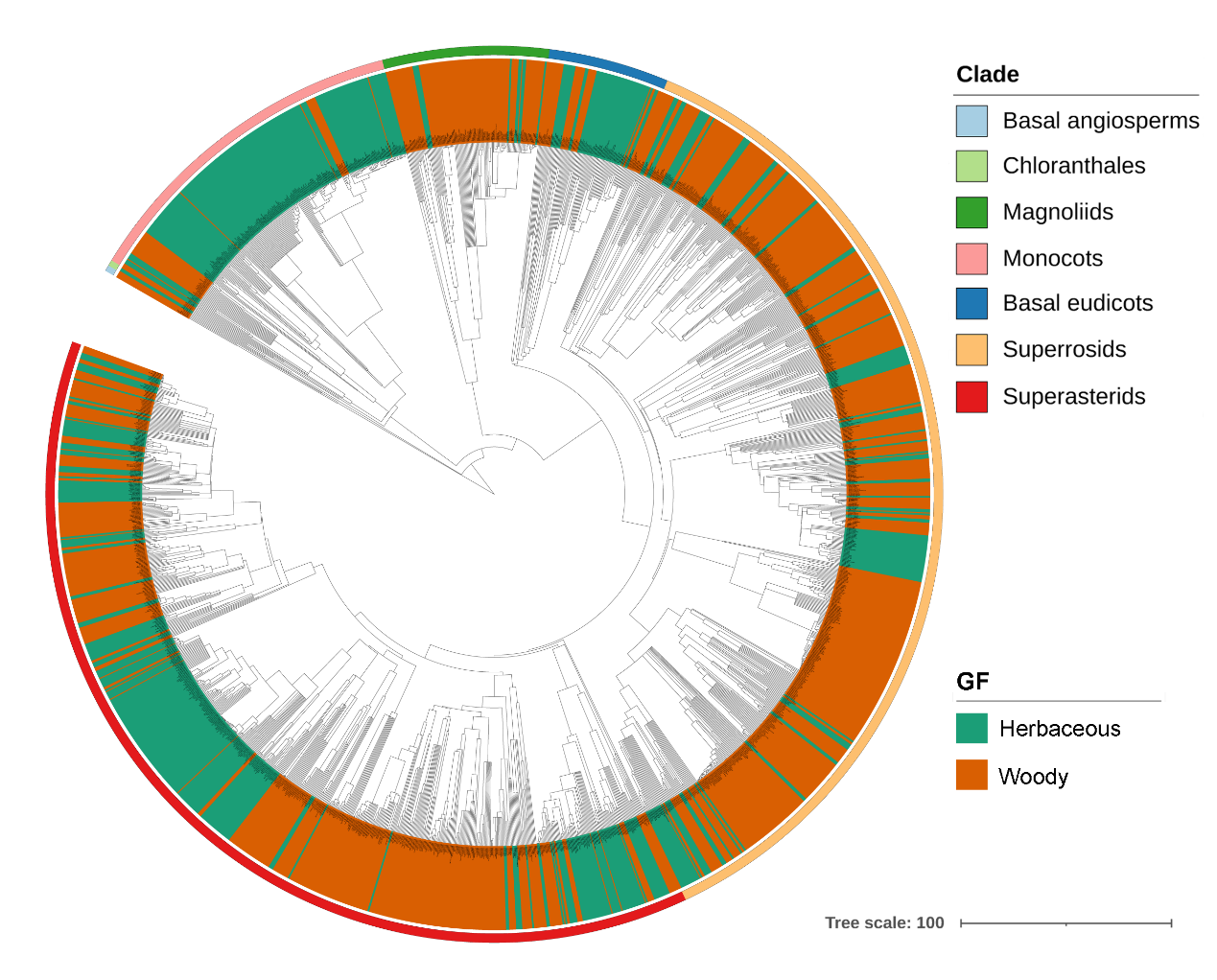


**Supplementary Figure 1** Phylogenetic tree of 1,819 angiosperm species at the species level in this study. Coloured bars around a phylogeny represent seven major phylogenetic clades, and the color on the tip label represents different plant growth forms.


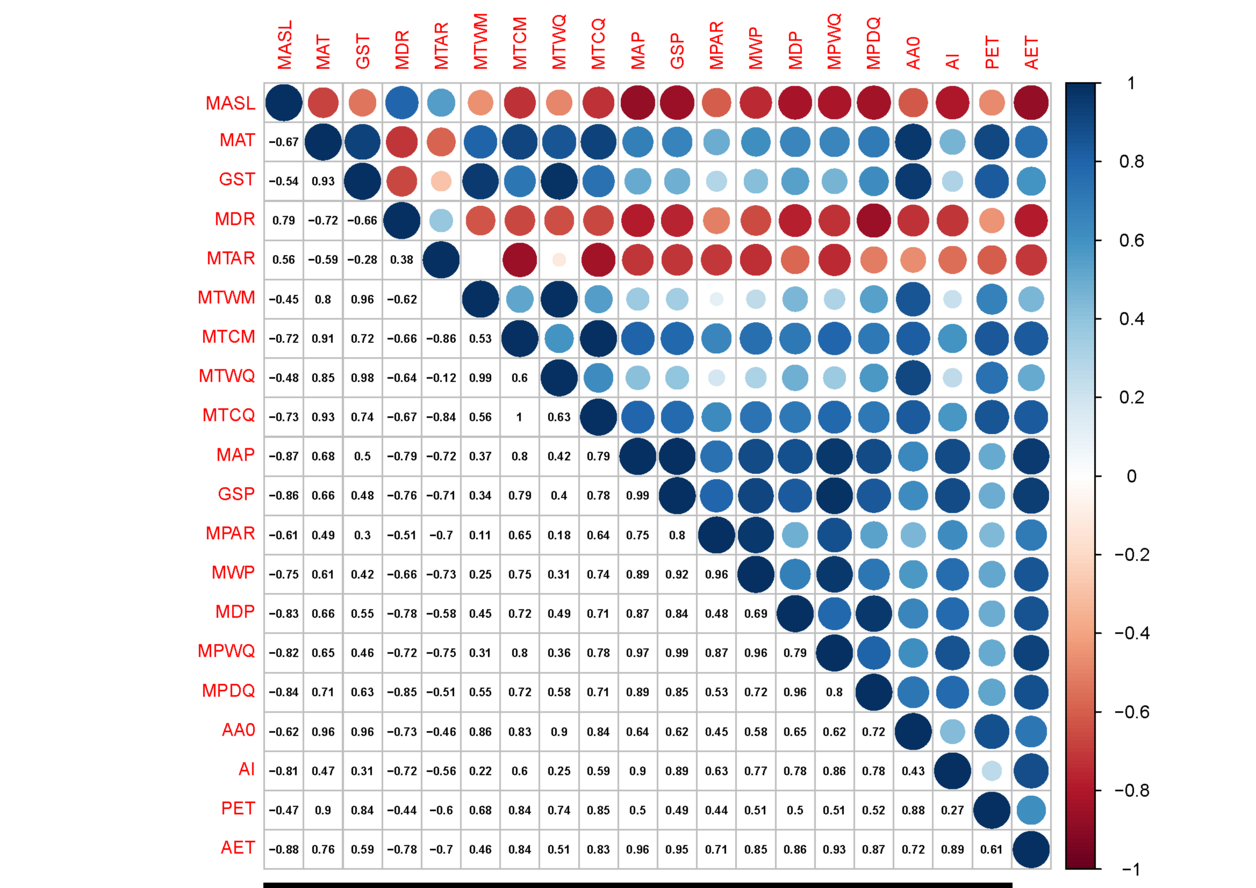


**Supplementary Figure 2** Correlation matrix among the 20 climate variables (*n* = 530). All variables were Yeo-Johnson transformed (see Table S2). The “blank” indicated no significant relationships between two climatic variables (*p* > 0.05). See Table S1 for abbreviations.


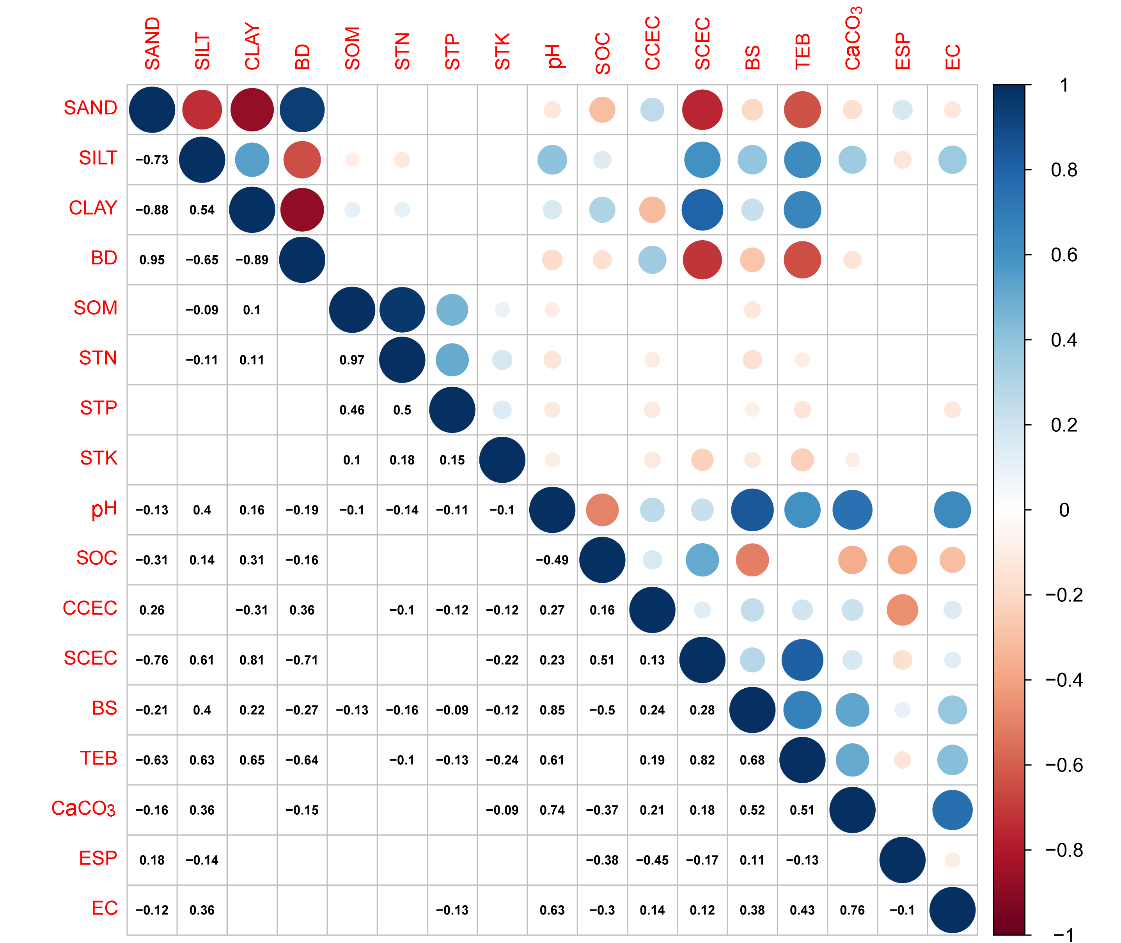


**Supplementary Figure 3** Correlation matrix among the 17 soil variables (*n* = 530). All variables were Yeo-Johnson transformed (see Table S2). The “blank” indicated no significant relationships between two soil variables (*p* > 0.05). See Table S1 for abbreviations.
