# Supplementary material for: Distinct Responses of Leaf Traits to Environment and Phylogeny Between Herbaceous and Woody Angiosperm Species in China
Source: Front Plant Sci. 2021 Dec 7;12:799401. doi: 10.3389/fpls.2021.799401 (PMC8688848; doi:10.3389/fpls.2021.799401)
Supplement: Supplementary file 2 [file Data_Sheet_2.docx]

**Supplementary data**

**Appendix A** – Literature used to extend the China Plant Trait Database (Wang et al., 2018)

An, H. (2012). Effects of grazing disturbance on leaf traits and their interrelationships of plants in desert steppe. *Chinese Journal of Applied Ecology* 23 (11), 2991-2996. doi: 10.13287/j.1001-9332.2012.0452

An, H., and Shangguan, Z.P. (2007). Photosynthetic characteristics of dominant plant species at different succession stages of vegetation on Loess Plateau. *Chinese Journal of Applied Ecology* 18 (6), 1175-1180. doi: 10.13287/j.1001-9332.2007.0198

Bai, K.D., Jiang, D.B., and Wan, X.C. (2013). Photosynthesis-nitrogen relationship in evergreen and deciduous tree species at different altitudes on Mao’er Mountain, Guangxi. *Acta Ecologica Sinica* 33, 4930-4938. doi: 10.5846/stxb201205200750

Bai, W.J., Zheng, F.L., Dong, L.L., and Ding, X.B. (2010). Leaf traits of species in different habits in the water-wind erosion region of the Loess Plateau. *Acta Ecologica Sinica* 30 2529-2540.

Cai, Z.Q., Schnitzer, S.A., and Bongers, F. (2009). Seasonal differences in leaf-level physiology give lianas a competitive advantage over trees in a tropical seasonal forest. *Oecologia* 161, 25-33. doi: 10.1007/s00442-009-1355-4

Chang, Y.N., Zhong, Q.L., Cheng, D.L., Xu, C.B., Hu, B., and Zhang, Z. (2013). Stoichiometric characteristics of C, N, P and their distribution pattern in plants of *Castanopsis carlesii* natural forest in Youxi. *Journal of Plant Resources and Environment* 22, 1-10. doi: 10.3969/j.issn.1674-7895.2013.03.01

Chen, C., Liu, D.H., Wu, J.J., Kang, M.Y., Zhang, J.T., Liu, Q.R., et al. (2015). Leaf traits of *Quercus wutaishanica* and their relationship with topographic factors in Mount Dongling. *Chinese Journal of Ecology* 34, 2131-2139. doi: 10.13292/j.1000-4890.2015.0174

Chen, F.Y., Luo, T.X., Zhang, L., Deng, K.M., and Tian, X.Y. (2006). Comparison of leaf construction cost in dominant tree species of the evergreen broadleaved forest in Jiulian Mountain, Jiangxi Province. *Acta Ecologica Sinica* 26, 2486-2493. doi: 10.3321/j.issn:1000-0933.2006.08.009

Chen, Z.C., Liu, C., Liu, X.J., and Wan, X.C. (2017). Effects of irradiation and tree size on the carbon-water balances of *Quercus aliena var. acuteserrata*. *Scientia Silvae Sinicae* 53, 18-25. doi: 10.11707/j.1001-7488.20170903

Cheng, J.H., Chu, P.F., Chen, D.M., and Bai, Y.F. (2016). Functional correlations between specific leaf area and specific root length along a regional environmental gradient in Inner Mongolia grasslands. *Funct. Ecol.* 30, 985-997. doi: 10.1111/1365-2435.12569

Dang, J.J., Zhao, C.Z., Li, Y., Hou, Z.J., and Dong, X.G. (2015). Relationship between leaf traits of *Melica przewalskyi* and slope aspects in alpine grassland of Qilian Mountains, China. *Chinese Journal of Plant Ecology* 39, 23-31. doi: 10.17521/cjpe.2015.0003

Deng, L., Wang, H.Z., Shangguang, Z.P., and Liu, G.Q. (2010). Variations of specific leaf area and nutrients of Chinese caragana in the Loess Plateau region suffering both wind and water erosions. *Acta Ecologica Sinica* 30, 4889-4897.

Dong, T.F., Feng, Y.L., Lei, Y.B., and Zhang, L.K. (2012). Comparison on leaf functional traits of main dominant woody species in wet and dry habitats. *Chinese Journal of Ecology* 31, 1043-1049.

Du, H.D. (2010) Ecological responses of foliar anatomical structure & physiological characteristics of dominant plants at different site conditions in north Shaanxi Loss Plateau. Northwest A&F University, Xi’an, China.

Du, N., Zhang, X.R., Wang, W., Chen, H., Tan, X.F., Wang R.Q. (2011). Foliar phenotypic plasticity of a warm-temperate shrub, *Vitex negundo var. heterophylla*, to different light environments in the field. *Acta Ecologica Sinica,* 20, 189-199.

Duan, Y.Y., J., S.L., Q., N.S., T., H., H., Y.G., and F., H.W. (2017). Variation in leaf functional traits of different-aged *Robinia pseudoacacia* communities and relationships with soil nutrients. *Chinese Journal of Applied Ecology* 28, 28-36. doi: 10.13287/j.1001-9332.201701.036

Feng, Q.H., Cheng, R.M., Shi, Z.M., Liu, S.R., Wang, W.X., Liu, X.L., et al. (2013). Response of leaf functional traits and the relationships among them to altitude of *Salix dissa* in Balang Mountain. *Acta Ecologica Sinica* 33, 2712-2718. doi: 10.5846/stxb201202020136

Feng, Q. H. (2011). The study on the response of foliar δ13C of different life from plants to altitude in subalpine area of Western Sichuan, China. Chinese Acedemy of Forestry, Beijng, China.

Gao, J., Xu, B., Wang, J.N., Zhou, H.Y., Wang, Y.X., Wu, Y. (2015). Correlations among leaf traits of typical shrubs and their responses to different light environments in shrub-grassland of southern China. *Chinese Journal of Ecology* 34, 2424-2431. doi: 10.13292/j.1000-4890.2015.0212

Gao, S.P., Li, J.X., u, M.C., Chen, X., and Dai, J. (2007). Leaf N and P stoichiometry of comm on species in successional stages of the evergreen broad-leaved forest in Tiantong National Forest Park, Zhejiang Province, China. *Acta Ecologica Sinica* 27, 947-952. doi: 10.3321/j.issn:1000-0933.2007.03.015

Guo, F.C. (2015). The photosynthetic characteristics of precious broad-leaved tree species in south subtropics and their relationships with leaf functional traits. Guangxi University, Nanning, China.

Guo, Q.Q., Luo, D.Q., Wang, S.L., and Li, J.R. (2013). Effect of forest gaps on leaf morphological and photosynthetic characteristics of main species in *Abies georgei Var. smithi* Forest. *Journal of Northeast Forestry University* 41, 48-51. doi: 10.13759/j.cnki.dlxb.2013.09.026.

Guo, W.J. (2015). Exploring the relationship between arbuscular mycorrhizal fungi and plants based on phylogeny and plant traits. Lanzhou University,

Hao, Z., Kuang, Y.W., and Kang, M. (2015). Untangling the influence of phylogeny, soil and climate on leaf element concentrations in a biodiversity hotspot. *Funct. Ecol.* 29, 165-176. doi: 10.1111/1365-2435.12344

He, C.X., Li, J.Y., Meng, P., and Zhang, J.S. (2013). Changes of leaf traits and WUE with crown height of four tall tree species. *Acta Ecologica Sinica* 33, 5644-5654. doi: 10.5846/stxb201304020586

He, J.S., Wang, Z.H., Wang, X.P., Schmid, B., Zuo, W.Y., Zhou, M., et al. (2006). A test of the generality of leaf trait relationships on the Tibetan Plateau. *New Phytol.* 170, 835-848. doi: 10.1111/j.1469-8137.2006.01704.x

He, Y.T. (2007). Studies of physioecological traits of 30 plant species in the Subalpine Meadow of the Qinling Mountains. The Chinese Academy of Sciences, Beijing, China.

Hou, M.M. (2017). Adaptive evolution of some species from Sedges (Carex: Cyperaceae) based on phylogeny and leaf functional traits to habitat in the Poyang Lake area. Nanchang University, Nanchang, China.

Hou, Y., Liu, M.X., and Sun, H.R. (2017). Response of plant leaf traits to microhabitat change in a subalpine meadow on the eastern edge of Qinghai-Tibetan Plateau, China. *Chinese Journal of Applied Ecology* 28, 71-79. doi: 10.13287/j.1001-9332.201701.024

Hu, Q.W., Zheng, L., Wu, Q., Li, X.F., Cao, Y., and Ding, M.J. (2010). Study on nitrogen and phosphorus content in the leaves of dominant plant species in sandy hills along Poyang Lake. *Ecological Sciences* 29, 97-101. doi: 10.3969/j.issn.1008-8873.2010.02.001

Hu, S.J., Wang, D.Y., Zheng, Y.L., Yan, X.H. (2016). Ecological adaptation of *Cypripedium flavum* at forest edge and in forest. *Journal of Chongqing Normal University (Natural Science)* 33, 137-141. doi: 10.11721/cqnuj20160605

Hu, W.Q. (2014). Study of structure of population and functional traits of *Archidendron clypearia* in Shimen National Forest Park. Chinese Academy of Forestry, Beijing, China.

Hu, W. X. (2011). The response of common species to fertilization based on plant traits in Tibetan Plateau alpine meadow. Lanzhou University, Lanzhou, China.

Huang, J.J., and Wang, X.H. (2003). Leaf nutrient and structural characteristics of 32 evergreen broad -leaved species. *Journal of East China Normal University (Natural Science)*, 92-97. doi: 10.3969/j.issn.1000-5641.2003.01.016

Huang, Y.L. (2015) The research about the turnover patterns and moisture adaptations mechanism of major species on the South-North-facing slope gradient. Lanzhou University, Lanzhou, China.

Jia, Q.Q. (2011). Functional traits of fine roots and their relationship with leaf traits of 50 major species in a subtropical forest in Gutianshan. Qiqihar University, Qiqihar, China.

Jiang, L.X., and Li, Y. (2008). Comparison on architecture characteristics of root systems and leaf traits for three desert shrubs adapted to arid habitat. *Journal of Desert Research* 28, 1118-1124. doi: 1000-694X(2008)06-1118-07

Jing, G.H. (2017). Responses of grassland community structure and functions to management practices on the semi-arid area of Loess Plateau. University of Chinese Academy of Sciences, Beijing, China.

Kang, H.J., Liu, P., Xu, G.D., Chen, Z.L., and Wei, F.M. (2008). Ecophysiological response of Emmenopterys henryi to the habitats in different altitudes in China’s Dapan Mountain Natural Reserve. *Journal of Plant Ecology (Chinese Version)* 32 (4), 865-872. [doi: 10.3773/j.issn.1005-264x.2008.04.015](https://doi.org/10.3773/j.issn.1005-264x.2008.04.015)

Kang, M. (2012). Spatial distribution pattern and its causes of woody plant functional traits in Tiantong region, Zhejiang Province. East China Normal University, Shanghai, China.

Li, F. (2011). Comparison of functional traits in semi-humid evergreen broad-leaved forest in Western Hill of Kunming. Yunnan University, Kunming, China.

Li, F.L., Bao, W.K., and Wu, N. (2007). Relationship between leaf traits in *Jasminum humile* and environmental factors in the Dry Valley of the Upper Minjiang River. *Journal of Tropical and Subtropical Botany* 15, 315-323. doi: 10.3969/j.issn.1005-3395.2007.04.007

Li, K., and Xiang, W.H. (2011). Comparison of specific leaf area, SPAD value and seed mass among subtropical tree species in hilly area of Central Hunan, China. *Journal of Central South University of Forestry &Technology* 31, 213-218. doi: 10.14067/j.cnki.1673-923x.2011.05.041

Li, L., McCormack, M.L., Ma, C.G., Kong, D.L., Zhang, Q., Chen, X.Y., et al. (2015). Leaf economics and hydraulic traits are decoupled in five species-rich tropical-subtropical forests. *Ecol. Lett.* 18, 899-906. doi: 10.1111/ele.12466

Li, L. (2015). The spatial and temporal stoichiometry characteristics of Machilus plantation of Huitong. Central South University of Forestry and Technology, Changsha, China.

Li, Q.J., Zhao, Z.R., Chen, W.X., Wang, M.J., Ding, S.T., and Wu, J.X. (2015). Changes in leaf traits of *Quercus liaotungensis Koidz* at different alititude in Kongtong Mountain of Gansu Province. *Journal of Lanzhou University (Natural Sciences)* 52, 13-19, 30. doi: 10.13885/j.issn.0455-2059.2015.01.003

Li, S.J., Su, P.X., Zhang, H.N., Zhou, Z.J. and Xie, T.T. (2013). Characteristics and relationships of foliar water and leaf functional traits of desert plants. *Plant Physiology Journal* 49, 153-160. doi: 10.13592/j.cnki.ppj.2013.02.011

Li, W.Q., Xu, Q., Li, J., Li, S.L., Yu, Q.C., and Zhang, Z.M. (2017). Quantification of ecotone width of returned forest land from farmland based on specific leaf area. *Journal of West China Forestry Science* 46, 117-121. doi: 10.16473/j.cnki.xblykx1972.2017.01.022

Li, X.L., Li, X.H., Jiang, D.M., Liu, Z.M., and Wang, H.M. (2005). Leaf morphological characters of 22 compositae herbaceous species in Horqin sandy land. *Chinese Journal of Ecology* 24, 1397-1401. doi: 10.13292/j.1000 -4890.2005.0169

Li, Y., Yao, J., Yang, S., and Hou, J.H. (2012). Trait differences research on leaf function of liaodong oak forest main species in Dongling mountain. *Guangdong Agricultural Sciences* 39, 159-162. doi: 10.16768/j.issn.1004-874x.2012.23.058

Li, Y., Zhao, C.Z., Dong, X.Q., Hou, Z.Q., Ma, X.L., and Zhang, Q. (2013). Responses of Stellera chamaejasme twig and leaf traits to slope aspect in alpine grassland of Northwest China. *Chinese Journal of Ecology* 32, 3145-3151. doi: 10.13292/j.1000-4890.2013.0483

Li, Y.H., Luo, T.X., Lu, Q., Tian, X.Y., and Yang, H.H. (2005). Comparisons of leaf traits among 17 major plant species in Shazhuyu Sand Control Experimental Station of Qinghai Province. *Acta Ecologica Sinica* 25, 994-999. doi: 10.3321/j.issn:1000-0933.2005.05.008

Li, Y.L., Meng, Q.T., Zhao, X.Y., and Cui, J.Y. (2008). Relationships of fresh leaf traits and leaf litter decomposition in Kerqin sandy land. *Acta Ecologica Sinica* 28, 2486-2492. doi: 10.3321/j.issn:1000-0933.2008.06.007

Li, Y.Y. (2015). The hydraulic drought-resistance strategies of *Salix psammophila* and *Caragana korshinskii* in water/wind erosion crisscross region of Northern Shaanxi. Northwest agricultural & Forestry University, Xi’an, China.

Li, Z. (2013). C, N and P stoichiometry of Suaeda salsa in coastal wetlands, China. Nanjing University, Nanjing, China.

Li, Z.F., Guo, P.J., Liu, W.S., and Zheng, Z. (2013). C, N and P stoichiometry of young trees in Montane Moist Evergreen Broad-Leaved Forest of Ailao Mountains. *Journal of Northeast Forestry University* 41, 22-26. doi: 10.13759/j.cnki.dlxb.2013.04.025

Liu, C.H., and Li, Y.Y. (2013). Relationships between leaf traits and PV curve parameters in the typical deciduous woody plants occurring in Southern Huanglong Mountain. *Journal of Northwest Forestry University* 28, 1-5. doi: 10.3969/j.issn.1001-7461.2013.06.01

Liu, G.F., Freschet, G.T., Pan, X., Cornelissen, J.H.C., Li, Y., and Dong, M. (2010). Coordinated variation in leaf and root traits across multiple spatial scales in Chinese semi-arid and arid ecosystems. *New Phytol.* 188, 543-553. doi: 10.1111/j.1469-8137.2010.03388.x

Liu, J.H., Zeng, D.H., and Don, K.L. (2006). Leaf traits and their interrelationships of main plant species in southeast Horqin sandy land. *Chinese Journal of Ecology* 25, 921-925. doi: 10.13292/j .1000 -4890.2006.0175

Liu, L.H. (2012). The traits and adaptive strategy of main herbaceous plant and lianas on micro-topographical units in Huangguangyu reserves of Anhui Province. East China Normal University, Shanghai, China.

Liu, W.D., Su, J.R., Li, S.F., Zhang, Z.J., and Li, Z.W. (2010). Stoichiometry study of C, N and P in plant and soil at different successional stages of monsoon evergreen broad-leaved forest in Pu’er, Yunnan Province. *Acta Ecologica Sinica* 30, 6581-6590.

Liu, X.Z., Feng, H.Y., Cai, C.J., Fan, S.H., and Liu, G.L. (2015). Response of leaf functional traits of Moso bamboo during the invading process into the broad-leaved forest. *Journal of Beijing Forestry University*, 37, 8-17. doi: 10.13332/j.1000-1522.20150157

Lu, J.Z., Miao, Y.M., Zhang, H.F., and Bi, R.C. (2010). Comparisons of leaf traits among different functional types of plant from Huoshan Mountain in the Shanxi Province. *Journal of Wuhan Botanical Research* 28, 460-465.

Luo, Q., Liu, H., Wu, G.L., He, P.C., Hua, L., Zhu, L.W., et al. (2018). Using functional traits to evaluate the adaptability of five plant species on tropical coral islands. *Acta Ecologica Sinica* 38, 1256-1263. doi: 10.5846/stxb201612152597

Lv, J.Z., Miao, Y.M., Zhang, H.F., and Cheng, B. R. (2010). Comparisons of leaf traits among different functional types of plant from Huoshan Mountain in the Shanxi Province. *Journal of Wuhan Botanical Research* 28, 460-465. doi: 10.3724/SP.J.1142.2010.40460

Ma, J., Wu, L.F., Wei, X., Ye, W.H., Huang, H.L., and Shen, H. (2015). Habitat adaptation of two dominant tree species in a subtropical monsoon forest: leaf functional traits and hydraulic properties. *Guihaia* 35, 261-268. doi: 10.11931/guihaia.gxzw201403019

Ma, J.M., Zhang, X.Z., Liang, S.C., Chen, T., and Huang, Q.J. (2012). Leaf traits of common plants in Yaoshan Mountain of Guilin, China. *Journal of Guangxi Normal University: Natural Science Edition* 30, 77-82. doi: 10.16088/j.issn.1001-6600.2012.01.020

Mi, Z.R., Huang, Y.Y., Gan, H.J., Zhou, W.J., Flynn, D.F.B., and He, J.S. (2015). Leaf P increase outpaces leaf N in an Inner Mongolia grassland over 27 years. *Biol. Lett.* 11, 5. doi: 10.1098/rsbl.2014.0981

Mo, J.M., Kong, D.Q., Huang, Z.L., Yu, Q.F., and Kong, G.H. (2000). Distribution pattern of nutrient elements in plants of Dinghushan Lower Subtropical Evergreen Broad-Leaved Forest. Journal of Tropical and Subtropical Botany 8, 198-206.

Niu, D.C., Li, Q., Jiang, S.G., Chang, P.J., and Fu, H. (2013). Seasonal variations of leaf C:N:P stoichiometry of six shrubs in desert of China’s Alxa Plateau. *Chinese Journal of Plant Ecology* 37, 317-325. doi: 10.3724/SP.J.1258.2013.00031

Niu, K.C., He, J.S., and Lechowicz, M.J. (2016). Grazing-induced shifts in community functional composition and soil nutrient availability in Tibetan alpine meadows. *J. Appl. Ecol.* 53, 1554-1564. doi: 10.1111/1365-2664.12727

Niu, S.L. (2004). Photosynthesis research on the predominant legume species in Hunshandak Sandland. The Chinese Academy of Science, Beijing, China.

Pan, H.L., Tian, Y., Liu, X.L., Cai, X.H., He, F., and Li, M.H. (2011). Responses of physiological and ecological traits of *Fargesia angustissima* to the altitudinal changes in Wolong Nature Reserve, Southwestern China. *Journal of Sichuan Forestry Science and Technology* 32, 25-30. doi: 10.16779/j.cnki.1003-5508.2011.01.004

Ren, Q.J., Li, H.L., and Bu, H.Y. (2015). Comparison of physiological and leaf morphological traits for photosynthesis of the 51 plant species in the Maqu alpine swamp meadow. *Chinese Journal of Plant Ecology* 39, 593-603. doi: 10.17521/cjpe.2015.0057

Rong, Q.Q., Liu, J.T., Xia, J.B., Lu, Z.H., and Guo, C.H. (2012). Leaf N and P stoichiometry of *Tamarix chinensis* L. in Laizhou Bay wetland, Shandong Province of East China. *Chinese Journal of Ecology* 31, 3032-3037. doi: 10.13292/j.1000-4890.2012.0400

Shang, K.K. (2011). Differentiation and maintenance of relict deciduous broad-leaved forest patterns along micro-topographic gradient in subtropical area, East China. East China Normal University, Shanghai, China.

Song, G. (2013). Adaptive strategies and responses of *Robinia Pseudoacaia* functional traits to environment in Loess Plateau, North Shaanxi, China. Northwest A&F University, Xi’an, China.

Song, Y.T. (2012). Study on functional plant ecology in Songnen Grassland, Northeast China. Northeast Normal University, Xi’an, China.

Su, W.H., Shi, Z., Yang, B., Yang, J.J., Zhao, G.H., and Zhou, R. (2015). Intraspecific functional trait variation in a tree species (*Lithocarpus dealbatus*) along latitude. *Plant Diversity and Resources* 37, 309-317. doi: 10.7677/ynzwyj201514107

Sun, K., Jiang, B.Y., Zhang, S.H., Hou, Q.Z., and Su, X. (2014). Study on the response of leaf size traits in *Hippophae tibetana schlecht* with precipitation and temperature. *Journal of Northwest Normal University (Nature Science)* 50, 71-76. doi: 10.16783/j.cnki.nwnuz.2014.06.015

Sun, S.C., Jin, D.M., and Li, R.J. (2006). Leaf emergence in relation to leaf traits in temperate woody species in East-Chinese *Quercus fabri* forests. *Acta Oecol.-Int. J. Ecol.* 30, 212-222. doi: 10.1016/j.actao.2006.04.001

Tan, X. Y. (2014). Research on leaf functional diversity of forest communities in rainy area of south-west China. Sichuan Agricultural University, Chengdu, China.

Tang, Q.Q. (2016). Variation in functional traits of plants in the subtropical evergreen and deciduous broad-leaved mixed forest. Chinese Academy of Forestry, Beijing, China.

Tang, Y. (2010). Inter-specific variations and relationships in leaf traits of major temperate species in northeastern China. Northeast Forestry University, Harbin, China.

Wang, B.B. (2015). A study on ecological stoichiometry of six kinds of dominant shrubs in Huangcangyu Nature Reserve. Huaibei Normal University, Huaibei, China.

Wang, H., Harrison, S.P., Prentice, I.C., Yang, Y.Z., Bai, F., Togashi, H.F., et al. (2018). The China Plant Trait Database: toward a comprehensive regional compilation of functional traits for land plants. *Ecology* 99, 500-500. doi: 10.1002/ecy.2091

Wang, J.Y., Zhang, H., Yu, M.K., Fang, Y.M., and Wu, T.G. (2017). Response of leaf traits of *Quercus acutissima* to environmental factors at regional scale. *Ecology and Environmental Sciences* 26, 754-762. doi: 10.16258/j.cnki.1674-5906.2017.05.005

Wang, J.Y., Wang, S.Q., Li, R.L., Yan, J.H., Sha, L.Q., and Han, S.J. (2011). C:N:P stoichiometric characteristics of four forest types’ dominant tree species in China. *Chinese Journal of Plant Ecology* 35, 587-595. doi: 10.3724/SP.J.1258.2011.00587

Wang, J.Y., Lan, J.C., Long, T., Xie, Y.J., Lu, X.M., Lei, L.Q., et al. (2013). Ecological stoichiometry characteristics of nitrogen, phosphorus and potassium in liana leaf of evergreen broad-leaf forest. *Journal of Southern Agriculture* 44, 815-818. doi: 10.3969/j:issn.2095-1191.2013.5.815

Wang, K.B. (2011). Vegetation ecological features and net primary productivity simulation in Yangou watershed in the loess hill-gully areas of China. University of Chinese Academy Sciences, Beijing, China.

Wang, S.S. (2016). The traits and adaptive strategy of main herbaceous plants and lianas on micro-topographical units in Longjishan reserves of Anhui Province. Huaibei Normal University, Huaibei, China.

Wei, J. (2014). Study on leaf traits of *Quercus baronii* in Yun Qiu mountain. Shanxi Normal University, Xi’an, China.

Wei, L.P. (2014). Variations in functional traits of main tree species along tree-crown in broad-leaved *Korean Pine* Forest in Jiaohe, Jilin Province. Beijing Forestry University, Beijing, China.

Wei, L.Y., and Shangguan, Z.P. (2008). Relation between specific leaf areas and leaf nutrient contents of plants growing on slope lands with different farming-abandoned periods in the Loess Plateau. *Acta Ecologica Sinica* 28, 2526-2534. doi: 10.1509/jimk.16.3.108

Wu, L.L. (2011). Variation in Quercus variables leaf traits in relation to environmental factors. Shanghai Jiao Tong University, Shanghai, China.

Wu, R.X. (2015). Growth and reproductive characteristics of *Thermopsis lanceolate* and its responses to management practices in alpine meadow of the Qinghai-Tibetan Plateau. Gansu Agricultural University, Lanzhou, China.

Wu, T.G., Wu, M., Liu, L., and Xiao, J.H. (2010). Seasonal variations of leaf nitrogen and phosphorus stoichiometry of three herbaceous species in Hangzhou Bay coastal wetlands, China. *Chinese Journal of Plant Ecology* 34, 23-28. doi: 10.3773/j.issn.1005-264x.2010.01.005

Wu, T.G., Chen, B.F., Xiao, Y.H., Pan, Y.J., Chen, Y., and Xiao, J.H. (2010). Leaf stoichiometry of trees in three forest types in Pearl River Delta, South China. *Chinese Journal of Plant Ecology* 34, 58-63. doi: 10.3773/j.issn.1005-264x.2010.01.009

Xiao, D. (2015). Effects of simulated nitrogen deposition on leaf functional traits of common species in natural Pinus tabulaeformis forest in Taiyue Mountain, Shanxi, China. Beijing Forestry University, Beijing, China.

Xie, Y.J. (2013). The characteristics of 20 dominant plant functional traits in evergreen broad-leaved forest in Daming Mountain Nature Reserve, Guangxi. Guangxi University, Nanning, China.

Yan, E.R., Wang, X.H., Guo, M., Zhong, Q., and Zhou, W. (2010). C:N:P stoichiometry across evergreen broad-leaved forests, evergreen coniferous forests and deciduous broad-leaved forests in the Tiantong region, Zhejiang Province, eastern China. *Chinese Journal of Plant Ecology* 34, 48-57. doi: 10.3773/j.issn.1005-264x.2010.01.008

Yang, S. (2017). The adaptive strategies of main herbaceous plants traits to different micro-topographical units in Dashushan Mountain, Hefei. Huaibei Normal University, Huaibei, China.

Yuan, S. (2011). Preliminary research on plant functional traits and the capability of carbon sequestration of major tree species in Changbai Mountain Area. Northeast Normal University, Harbin, China.

Zhan, Y. Y. (2010). Study on the leaf functional traits of Davidia involucrate at the Houhe Nature Reserve in Hubei. Beijing Forestry University, Beijng, China.

Zhang, J.G., Fu, S.L., Wen, D.Z., and Zhang, L.L. (2009). Relationships of key leaf traits of 16 woody plant species in Low Subtropical China. *Journal of Tropical and Subtropical Botany* 17, 395-400.

Zhang, J.L., and Cao, K.F. (2009). Stem hydraulics mediates leaf water status, carbon gain, nutrient use efficiencies and plant growth rates across dipterocarp species. *Funct. Ecol.* 23, 658-667. doi: 10.1111/j.1365-2435.2009.01552.x

Zhang, J.L., Poorter, L., and Cao, K.F. (2012). Productive leaf functional traits of Chinese savanna species. *Plant Ecol.* 213, 1449-1460. doi: 10.1007/s11258-012-0103-8

Zhang, J.Y. (2008). Comparative study on the different plant functional groups leaf traits at the Maoershan Region. Northeast Forestry University, Harbin, China.

Zhang, M., Liu, F.D., An, S.Q., and Ou, X.Y. (2017). Comparison of leaf traits of the saplings of different structural and functional species in Tropical Montane Rain Forest. *Journal of Northeast Forestry University* 45, 13-17, 23. doi: 10.13759/j.cnki.dlxb.2017.09.003

Zhang, X.F. (2012). Leaf traits and relationships variations in two Quercus species along an altitudinal gradient in Qinling Mountain. Northwest University, Xi’an, China.

Zhao, A.J. (2014). Research on leaf functional diversity of forest communities in rainy area of southwest China. Sichuan Agricultural University, Chengdu, China.

Zhao, M.X. (2012). Analysis on the strategies of utilization of nitrogen and phosphorus in *Phragmites australis* and *Spartina alterniflora* in Chongming Dongtan Wetland based on ecological stoichiometry. East China Normal University, Shanghai, China.

Zheng, S.X., and Shangguan, Z.P. (2007). Spatial patterns of photosynthetic characteristics and leaf physical traits of plants in the Loess Plateau of China. *Plant Ecol.* 191, 279-293. doi: 10.1007/s11258-006-9242-0

Zheng, S.X., and Shangguan, Z.P. (2007). Photosynthetic physiological adaptabilities of *Pinus tabulaeform* and *Robinia pseudoacacia* in the Loess Plateau. *Chinese Journal of Applied Ecology* 18, 16-22. doi: 10.1360/yc-007-1324

Zheng, X.J., Li, S., and Li, Y. (2011). Leaf water uptake strategy of desert plants in the Junggar Basin, China. *Chinese Journal of Plant Ecology* 35, 893-905. doi: 10.3724/SP.J.1258.2011.00893

Zheng, Y.M. (2014). Carbon, nitrogen and phosphorus stoichiometry of plant and soil in the Sandy Hills of Poyang Lake. Jiangxi Normal University, Nanchang, China.

Zheng, Z.X. (2010). Comparison of plant leaf, height and seed functional traits in dry-hot valleys. Yunnan University, Kunming, China.

Zhong, C. L. (2016). Responses of element stoichiometry characteristics of Casuarina equisetifolia to distance from the coastline. Fujian Normal University, Fuzhou, China.

Zhou, J.Y., He, J.J., Guo, Z.Y., Wang, B.B., Zhang, X.W., and Guo, C.Y. (2010). A study on specific leaf area and leaf dry matter content of five dominant species in Xiangshan Mountain, Huaibei City, Anhui Province. *Journal of Huaibei Normal University (Natural Science)* 34, 51-54. doi: 10.3969/j.issn.2095-0691.2013.03.013

Zhou, X., Zuo, X.A., Zhao, X.Y., Liu, C., and Lu, P. (2015). Plant functional traits and interrelationship of 34 plant species in South Central Horqin Sandyland, China. *Journal of Desert Research* 35, 1489-1495. doi: 10.7522/j.issn.1000-694X.2014.00117

Zhu, B.R., Xu, B., and Zhang, D.Y. (2011). Extent and sources of variation plant functional traits in grassland. *Journal of Beijing Normal University (Natural Science)* 47, 485-489.

Zhu, H.X., Fu, B.J., Wang, S., Zhu, L.H., Zhang, L.W., Jiao, L., et al. (2015). Reducing soil erosion by improving community functional diversity in semi-arid grasslands. *J. Appl. Ecol.* 52, 1063-1072. doi: 10.1111/1365-2664.12442

Zhu, X.B., Liu, Y.M., and Sun, S.C. (2005). Leaf expansion of the dominant woody species of three deciduous Oak forests in Nanjing, East China. *Acta Phytoecologica Sinica* 29, 128-136. doi: 10.17521/cjpe.2005.0017
